# The Assessment of Competency in Thoracic Sonography (ACTS) scale: validation of a tool for point-of-care ultrasound

**DOI:** 10.1186/s13089-017-0081-0

**Published:** 2017-11-22

**Authors:** Scott J. Millington, Robert T. Arntfield, Robert Jie Guo, Seth Koenig, Pierre Kory, Vicki Noble, Haney Mallemat, Jordan R. Schoenherr

**Affiliations:** Division of Critical Care, University of Ottawa, 501 Smyth Road, Box 207, Ottawa, ON K1H 8L6 Canada

**Keywords:** Point-of-care, Thoracic ultrasound, Assessment tools, Education

## Abstract

**Background:**

The rapid adoption of point-of-care ultrasound (POCUS) has created a need to develop assessment tools to ensure that learners can competently use these technologies. In this study, the authors developed and tested a rating scale to assess the quality of point-of-care thoracic ultrasound studies performed by novices. In Phase 1, the Assessment of Competency in Thoracic Sonography (ACTS) scale was developed based on structured interviews with subject matter experts. The tool was then piloted on a small series of ultrasound studies in Phase 2. In Phase 3 the tool was applied to a sample of 150 POCUS studies performed by ten learners; performance was then assessed by two independent raters.

**Results:**

Evidence for the content validity of the ACTS scale was provided by a consensus exercise wherein experts agreed on the general principles and specific items that make up the scale. The tool demonstrated reasonable inter-rater reliability despite minimal requirements for evaluator training and displayed evidence of good internal structure, with related scale items correlating well with each other. Analysis of the aggregate learning curves suggested a rapid early improvement in learner performance with slower improvement after approximately 25–30 studies.

**Conclusions:**

The ACTS scale provides a straightforward means to assess learner performance. Our results support the conclusion that the tool is an effective means of making valid judgments regarding competency in point-of-care thoracic ultrasound, and that the majority of learner improvement occurs during their first 25–30 practice studies.

## Background

The term point-of-care ultrasound (POCUS) refers to a goal-directed ultrasound (US) exam performed directly by the treating physician in order to answer a well-defined question relevant to the immediate care of a patient. In certain clinical circumstances, POCUS has been shown to improve clinical outcomes (as in penetrating thoracic trauma; [1]), to increase patient safety (as with the insertion of central venous catheters; [2]), and to improve diagnostic accuracy over current standards of care (as in the detection of pneumothorax; [3]). A recent international statement written by experts from twelve critical care societies agreed that POCUS should be mandatory in the training of critical care physicians [4]. Despite the increased interest, methods for fostering development of these competencies are highly variable between centers with little agreement on how to train and assess learner proficiency [5].

One method for improving consistency is the development of assessment instruments. The development of an instrument requires that a group of experts come together to formalize their knowledge in a given domain by identifying features that they believe are related to competency. By examining learner performance over a number of training sessions, we can test to see whether experts are correct in their assumptions about the facets of a competency. Once an assessment instrument has been formalized, it can be used to monitor the development of learner competency and evaluate whether an educational intervention is effective at increasing proficiency. It would also support quality assurance and patient safety initiatives in general by ensuring that scan quality is adequate.

### Ultrasound competency and its development

Thoracic ultrasound represents a novel and interesting instrument in the POCUS toolkit [6]. It can, for example, help make the distinction between such clinically similar entities as cardiogenic and non-cardiogenic pulmonary edema [7]. Whereas most other ultrasound subtypes were developed by other specialist groups and later modified for use by acute care physicians, thoracic ultrasound was created and studied primarily by physicians in intensive care units and emergency departments. This fact, coupled with the relatively short history of thoracic ultrasound, means that the modality has been relatively understudied from an educational point of view.

The development of assessment instruments for POCUS has precedents. Using similar methods, we previously developed a scale to assess cardiac POCUS competency [8], demonstrating that cardiac ultrasound competency was associated with two main domains: Image Generation and Image Interpretation. Image Generation reflects a competency involving a learner’s knowledge of anatomy in combination with their visuospatial reasoning abilities. In contrast, while Image Interpretation requires that images have been generated successfully, it additionally necessitates clinical knowledge and diagnostic experience. Subsequently, we deployed that same tool to map out the typical cardiac ultrasound learning curves for novices [9]. For these reasons, the adoption of a similar approach in the context of thoracic ultrasound appears principled.

In the present study, we develop a scale capable of rapidly assessing the quality of point-of-care thoracic scans that requires minimal training in its application. As in our previous studies that examined cardiac POCUS we focused on construct validity, defined as the extent to which the feature of a test or scale can adequately measure what it purports to measure [10–13]. We use Messick’s [12] validation framework to provide evidence for the ACTS scale’s ability to measure the underlying features of POCUS competency. In the context of the present study, we first consider whether experts can reach a consensus on the features of competency in lung ultrasonography and whether multiple independent raters reliably produce similar ratings using the ACTS instrument (content evidence). We then consider how learners' performance for each component of the ACTS scale is related or unrelated to one another (internal structure evidence). For instance, scores obtained from subscales for proximate positions should be strongly related to one another whereas scores obtained from subscales for distal positions should be weakly or unrelated to one another. Finally, as we have developed the ACTS scale to be able to detect changes in competency over the course of training, we should obtain a learning curve that shows evidence of improvements in performance from earlier to later stages of training (response process evidence).

## Methods

### Phase 1: Developing the ACTS assessment tool

The ACTS scale content was developed in a series of three structured teleconferences with POCUS experts from across North America, most of whom had participated in the development and validation of our previous cardiac tool [8]. These teleconferences identified a comprehensive list of features and dimensions that define lung ultrasound competency (thematic saturation), considered what constitutes both perfect and minimally acceptable performance for thoracic POCUS (standard setting), and identified possible obstacles to implementing the use of lung ultrasound scale.

**Fig. 1:**
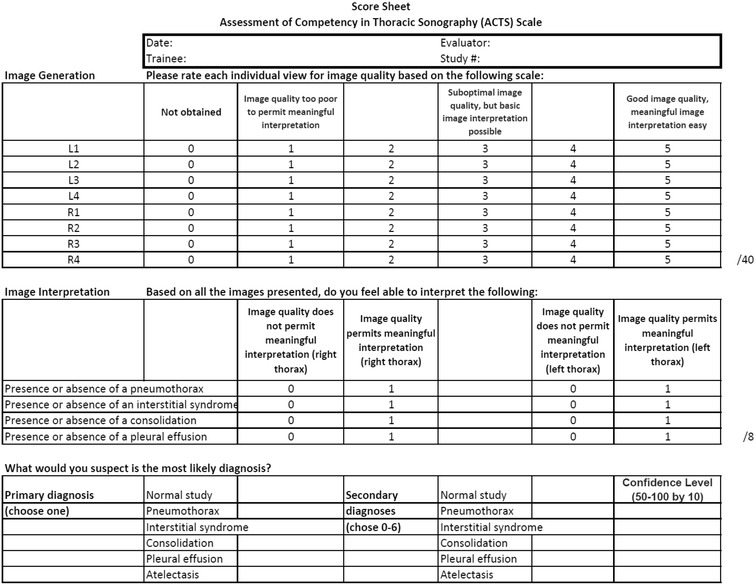
The Assessment of Competency in Thoracic Sonography (ACTS) tool

**Fig. 2:**
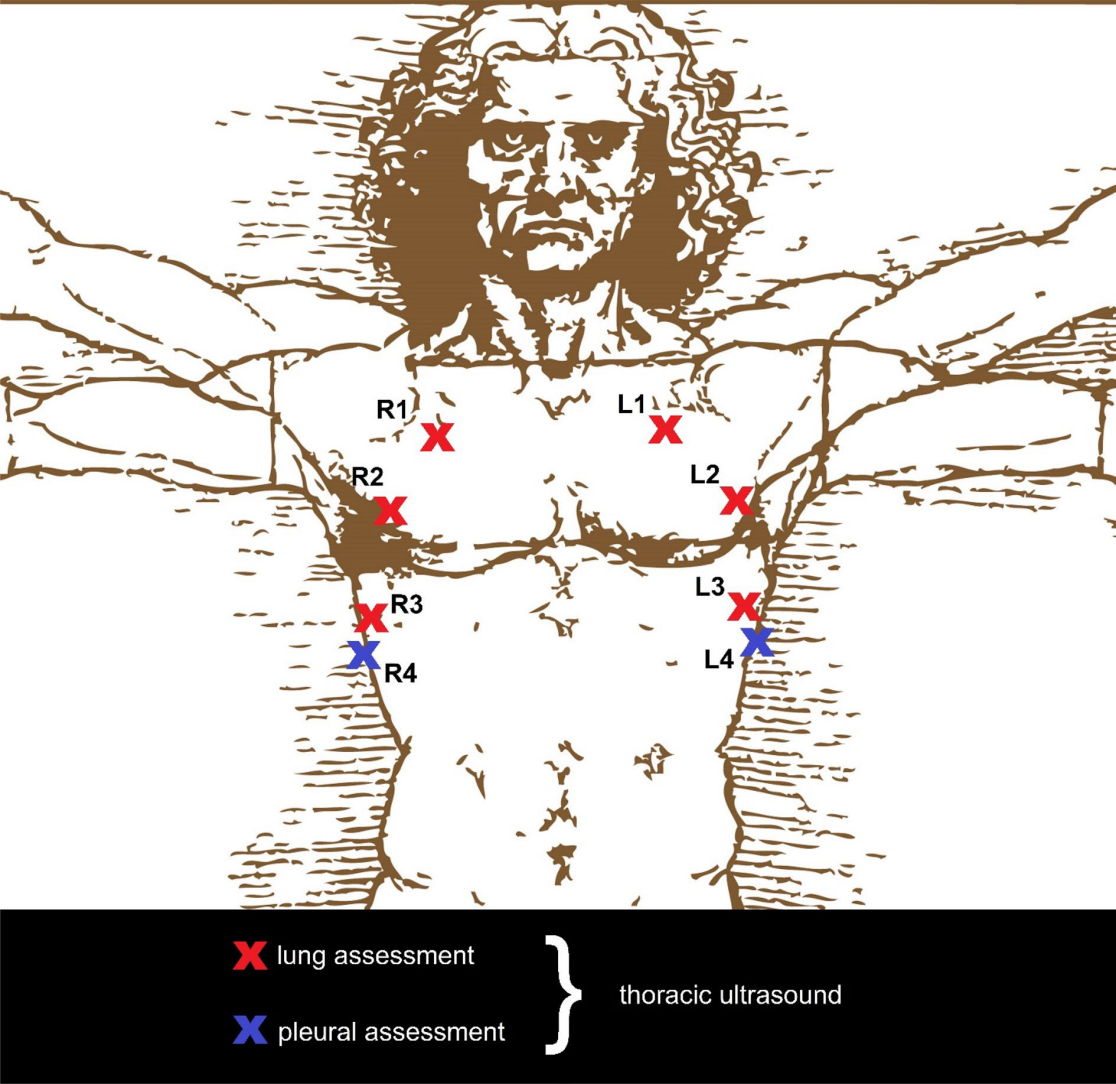
The eight common view for thoracic ultrasound

Following the approach of our previous scale [8], the ACTS tool (Fig. 1) divides assessment into two domains: (1) Image Generation and (2) Image Interpretation (Fig. 2). Image Generation subscale assesses image quality for each of the 8 typical thoracic views on a 6-point scale. Image Interpretation subscale uses a binary pass/fail assessment to decide whether an expert is able to assess each of the 4 common thoracic pathologies (pneumothorax, interstitial syndrome, consolidation, and pleural effusion) based on the images provided.

Additionally, we asked the experts to interpret the images (in absence of any clinical information), providing their opinion on the most likely diagnosis, any secondary diagnoses, and their relative confidence in making these judgments.

### Phase 2: Piloting the tool

A pilot ACTS tool was circulated to the same group of six experts and modified based on their feedback. A small series of POCUS studies were then tested by selecting a random sample of ten thoracic exams from the London Health Sciences Center database (London, ON, Canada), removing patient identifiers, and distributing them electronically. Ethics approval was granted by the London Research Ethics Board. After the same ten studies had been scored by each expert, a video conference call was held to review each study in an effort to improve the tool as well as to standardize the criteria used to judge the studies.

### Phase 3: Tool validation

Thoracic ultrasound studies produced by a group of ten POCUS learners at Western University in London, ON were selected for use in the study. The learners consisted of physician trainees in either emergency medicine or critical care, and were selected based on the criteria of: (1) having had basic training in POCUS, (2) currently participating in the local POCUS training program in London, ON, and (3) having recorded 50 thoracic POCUS studies as part of the local training program. All ultrasound images were stored in the local archiving system (Qpath Software, Telexy Healthcare, Port Coquitlam, BC). A series of representative video clips were extracted by taking the set of available US studies from each learner and sampling their portfolio of thoracic studies at regular intervals. These studies were then anonymized (with both patient and operator information removed), merged into the larger pool of sampled studies from all learners, randomized, and then distributed to the experts for evaluation using the ACTS tool. Each study was evaluated by a pair of experts and all ratings were subsequently pooled for statistical analysis.

### Statistical analysis

To investigate the validity of the ACTS scale, we reduced the dataset by obtaining average scores and their standard deviations (Table 1, discussed below). For our analysis of the content of the ACTS Scale, we examined inter-rater reliability between experts when assessing the US studies. High levels of inter-rater reliability would suggest that the ACTS scale is being used in an equivalent manner by raters, and is thus used by multiple raters in a similar manner. Second, we assessed the internal structure of the scale by examining how the eight ACTS scale items were related (Table 2). Our assumption that POCUS competency might be defined by two underlying factors (Image Generation and Image Interpretation) would be supported if ratings of items within a subscale were highly correlated. Finally, learners’ POCUS competency should improve over time. If the average learner performance increased from earlier to later stages of training, this would provide evidence that the ACTS scale adequately assesses the response process of learners. Taken together, multiple sources of evidence go toward demonstrating the validity of the ACTS Scale.

**Table 1:** Descriptive statistics for standardized scores on ACTS subscales for both the right and left analyses

ACTS scale item	Rating (*L*)			Rating (*R*)		
	Mean	Range	SD	Mean	Range	SD
V1	.8173	0–5	.235	.7853	0–5	.278
V2	.7873	0–5	.257	.6673	0–5	.381
V3	.6540	0–5	.320	.6040	0–5	.358
V4	.5993	0–5	.363	.5567	0–5	.387
Pneumothorax	.9500	0–1	.218	.8867	0–1	.318
Interstitial syndrome	.9300	0–1	.256	.8967	0–1	.305
Consolidation	.7067	0–1	.456	.6967	0–1	.460
Pleural effusion	.7367	0–1	.441	.7600	0–1	.428

Ratings are provided in rescaled values (.0–1.0) whereas the range is reported using the range provided on subscale used to assess learners (i.e., 0–5 and 0–1)

**Table 2:** Inter-item correlations for mean learner RACE ratings collapsed across raters

	1	2	3	4	5	6	7	8
V1_IG	–							
V2_IG	.415**	–						
V3_IG	.181**	.486**	–					
V4_IG	.088	.290**	.639**	–				
Pneumothorax	.487**	.274**	.143*	.042	–			
Interstitial syndrome	.417**	.351**	.322**	.239**	.554**	–		
Consolidation	.183**	.251**	.584**	.559**	.182**	.385**	–	
Pleural effusion	.132*	.190**	.618**	.572**	.157*	.354**	.778**	–

* *p* < .05

** *p* < .01

## Results

### Content validation and content experts

Evidence for the validity of the content of the ACTS tool was examined in terms of the extent to which experts agreed on the content of the rating scales and the extent to which raters provided comparable ratings on those scales. The structured interviews and subsequent expert discussions revealed that there appeared to be widespread consensus regarding POCUS in general and thoracic POCUS specifically. There was also consensus on Image Generation and Image Interpretation as the two broad determinants of basic thoracic POCUS competency, with the skill set of clinical integration being a necessary but more advanced skill.

### Raters and scale reliability

In order to examine the reliability of the ACTS scale, we assessed the extent to which each pair of expert raters produced similar responses (inter-rater reliability). Raters differed for each learner and for each training session. A sample of 150 observations was obtained, by taking fifteen studies from each of the ten learners. These ratings were randomly sampled over the course of the training sessions in order to model an overall learning curve (see below). Given that the Image Generation and Image Interpretation subscales are scored differently (using a 6-point and binary scale, respectively), ratings were rescaled to a range of .0–1.0 in order to compare the two subscales (e.g., items on the Image Interpretation scale that were rated as 0, 1, 2, 3, 4, and 5 would be rescaled to values of .0, .2, .4, .6, .8, and 1.0).

Table 1 presents the basic descriptive statistics for the ACTS subscales, with a few critical results. First, raters used the full scoring range of each of the subscales. Second, comparable values were obtained for both left and right images for the Image Generation subscale items suggesting that learners' performance was nearly equivalent in generating left- and right-sided images. Third, learners received higher ratings for Image Interpretation items for left-side images relative to right-side images, suggesting that right-side images might be more difficult for experts to interpret or for learners to perform.

In order to assess the reliability of the scale, we examine the scores provided by the two raters assigned to each case for each of the learners. The resulting Cronbach’s Alpha (*α* = .791) obtained by comparing overall standardized ratings for each learner reflects an acceptable overall level of inter-rater reliability for the ACTS scale as a whole. An assessment of individual subscales revealed that items assessing Image Generation produced a good degree of agreement between raters (*α* = .824) whereas Image Interpretation had an acceptable degree of agreement between raters (*α* = .709).

### Internal scale structure

While the previous analysis of correlations between scores from expert raters suggests that the scale was used in a consistent manner across different raters, it leaves unexamined the relationship between the 12 individual scale items (8 Image Generation and 4 Image Interpretation). After average across raters, the mean score for each of the 12 items was included in an inter-item correlation analysis. This analysis was conducted in order to examine whether ACTS items that should logically be related (e.g., imaging Lung View 1 and Lung View 2) will exhibit a stronger correlation than those items that are not as closely related (e.g., imaging Lung View 1 and Lung View 4).

Image Generation subscale items (items 1 through 8) and Image Interpretation subscale items (items 9 through 12) were included together in a correlational analysis. As Table 2 demonstrates, there is evidence that related items tend to strongly and positively correlate with one another. A critical outcome of the ACTS scale is evidenced in the correlations between items in the Image Generation subscale that measured a learner’s ability to generate images. Notably, the strength of the correlation decreased as a function of distance from one imaging location to another, as would be expected. We also found that the presence of pleural effusion was correlated with the image quality for viewing positions 3 and 4. As these locations are typical sites where clinicians investigate pleural effusion, it provides additional support for the validity of the ACTS scale.

### Learning process and construct validity

To assess whether learners’ POCUS competence increased over training, we examined whether there was evidence of change in performance over the course of training. A mean was obtained for each training sessions if available (i.e., the random sampling of learner performance did not always produce ratings for the same sessions for each learner).

**Fig. 3:**
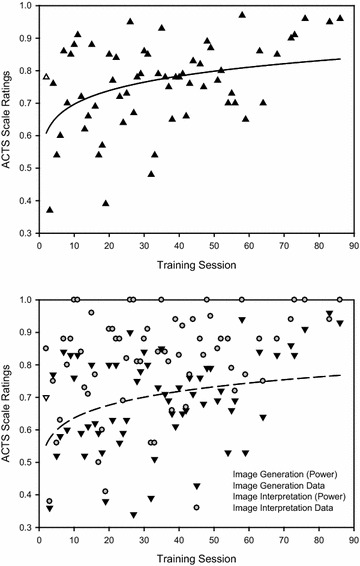
Learning curves for mean overall performance across ACTS subscales (top) and Image Generation and Image Interpretation subscales (bottom). Best-fit power functions are plotted in each

First, we examined learning for the overall ACTS score for each ultrasound study collapsing across mean score across subscale components. We selected models into examine whether (1) learning increased systematically over the course of the period of observation (linear function), (2) learning increased rapidly during initial phases of training and stabilized thereafter (power function), and (3) learning proceeded more slowly during initial phases of training, rapidly increased, and then reached a performance asymptote (sigmoid function; where learners improved up to a certain point and then reached a plateau). The three models fit for ACTS scale for overall performance are presented in Fig. 3. While each of the models provided a comparable fit, the power function provided the best fit. This indicates that learners’ performance increased rapidly throughout the early stages of in training (roughly sessions 1–25) but that later stages of training (beyond session 35) produced only moderate improvements in performance.

Second, given the observed differences in the Image Generation and Image Interpretation subscales, we analyzed participants learner curves for each of the subcomponents separately (Table 3). For the Image Generation subscale, slightly better fits were obtained for both power and sigmoid functions. Conforming to the results of our previous study examining POCUS competency development [9], this might suggest that learners benefit from early training, but after a sufficient number of session their performance no longer improves. Given that the power function provided an equivalent fit to the sigmoid function while using fewer parameters^1^, this suggests that the power function reflects a more conservative model of competency development. For the Image Interpretation subscale, the power function also offered the best fit, again suggesting that the abilities that underpin this portion of the scale improve with early training efforts. Thus, given the superior fits of the power functions in the case of both Image Generation and Image Interpretation results, this suggests that the largest effect occurs very early in training (sessions 1–25) and decreases but continued thereafter (beyond session 35). However, additional studies with more learners will need to be conducted to examine the generalizability of this finding.

**Table 3:** *R*-squared measures for curve fits for mean performance for all participants

	Image Generation score	Image Interpretation score	Overall score
Linear	.1696 (.133)	.1402 (.139)	.1816 (.123)
Power (2)	.2156 (.132)	.2156 (.130)	.1935 (.124)
Sigmoid (4)	.2170 (.131)	.1403 (.142)	.1976 (.124)

Standard error of the estimate is presented in parentheses

## Discussion

In this study we developed the ACTS scale, a rapid scoring instrument for lung sonography which can be applied while directly supervising a learner or while reviewing ultrasound scans after they are captured by an image archiving system. Based on our expert interviews, and building on our previous cardiac ultrasound tool [8], we decided that the early priority was to assess the learners’ ability to generate images of acceptable quality as this is the foundational step required to develop POCUS competency. As such, the scoring system was developed to capture both the image quality of the individual views (Image Generation domain) and the ability of an expert to make a clinical judgment based on the images provided (Image Interpretation domain).

There are several lines of evidence that provide support for the validity of the ACTS tool, including the high degree of consensus achieved within group of experts assembled. The group of six experts who evaluated the studies received a minimal amount of training, yet still achieved a good level of inter-rater reliability, suggesting that the tool can be used in a wide variety of clinical settings. While individual assessment instruments must be developed based on the demands of the particular clinical environment, ACTS appears to be an effective instrument to monitor the development of competency in lung ultrasonography.

There were strong, positive correlations observed within the measures of the Image Generation domain as well as similar trends within the measures of the Image Interpretation domain. The correlations between items within each of the subscales suggest that these assessment dimensions both make contributions to POCUS competency. Predictably, within the Image Generation subscale, images that were taken closer together (for example View 1 and View 2) were defined by much higher correlations than those images taken further apart (such as View 1 and View 4). This finding also supports the validity of the elements measured by the ACTS tool.

The correlations which were particularly strong can be interpreted clinically in a straightforward manner. For example, the strong correlation seen between the diagnosis of a pneumothorax and the quality of the R1 and L1 images follows from the fact that this condition is usually detected by placing in the ultrasound probe in those positions in typical clinical practice. Similarly, the detection of a pleural effusion correlated well with image quality ratings in the 3rd and 4th positions. Given that these are the typical anatomic locations where clinicians investigate for effusions, it provides further support for ACTS ability to identify lung sonography competency. Moreover, the absence of correlations between certain view positions and the presence of certain pathophysiologies provide additional support. For example, strong correlation between pneumothorax and the 4th scanning position or consolidation and the 1st scanning position would not be expected based on thoracic anatomy, and were not found in our study.

Our curve fitting analysis suggested that learning occurred throughout the course of the training sessions. In comparing three possible models of learning (linear, power, and sigmoid), we observed that a power model provided the best fit for the overall ACTS score as well as the scores for individual subscales. This suggests that learners rapidly acquired lung ultrasonography competencies earlier in training (sessions 1–25) but that competency development leveled off (after approximately session 35). This suggests that while additional training might be beneficial for learners, educators might wish to introduce additional training techniques or change the nature of the feedback provided to learners following 30 practice ultrasound studies. For instance, educators might wish to include simulation that includes diagnostic and treatment elements. Compared to our cardiac assessment tool [8], learners performed better with thoracic ultrasound early in their training. One possible explanation is that the small sample size or the fact that all learners were from a single center yielded an unrepresentative sample. For instance, there might be a self-selection bias wherein learners had better existing visuospatial abilities. Alternatively, previous US experience (cardiac and abdominal ultrasound) might have increased performance in the thoracic POCUS study conducted here. Future studies will need to use more participants and control for individual differences in general and specific clinical experience and knowledge.

Our study has other limitations that should be acknowledged. Given that evidence for validity was obtained in only one context, further work is needed to ensure that the tool is generalizable to other environments. Given that the study was conducted with learners in London, ON, it is entirely possible that the joint influence that educators have on the curriculum and the instrument would make it easier for learners to acquire the specific skills measured by ACTS. The fact that experts were drawn from a number of institutions suggests that this possibility is unlikely, however, it might be the case that the small number of experts (6) lead to a bias sample. As there continues to be disagreement in lung ultrasonography in general [14], this remains a real possibility. Our study assessed only expert ratings and did not incorporate any interpretation data from the learners who generated the actual studies; such a comparison would have been most interesting but was unfortunately unavailable to us. Finally, in our study the binary Image Interpretation subscale performed less well than Image Generation subscale, which was based on a 6-point rating scale. The binary scale was chosen for convenience, but it is certainly possible that lower reliability scores reflect the general disadvantage of checklists relative to general rating scales [15]. An assessment tool based on a rating scale for Image Interpretation as well as Image Generation would might have performed better, albeit at the cost of increased complexity.

Future studies should ideally compare the ACTS scale to other assessment methods such as the Objective Structured Clinical Examination or the Mini Clinical Examination to examine the relationship of our instrument to other instruments (providing evidence for relationship to other variables) as well as the consequences of implementing POCUS assessment on educators and learners (providing evidence of consequences; [12]). A larger study involving multiple centers would also help to allay concerns about generalizability.

## Conclusions

Overall, the results of the present study support the use of the ACTS scale to assess learners’ competency in point-of-care thoracic ultrasound, and suggests that the majority of learning occurs during the first 25–30 practice studies.

## Abbreviations

POCUS: point-of-care ultrasound
ACTS: Assessment of Competency in Thoracic Sonography

## Footnotes

1 The more parameters that are included in a model, the more likely it will fit the data. Models with fewer parameters are therefore preferred on the grounds of parsimony.
